# Nonlinear Topological Effects in Optical Coupled Hexagonal Lattice

**DOI:** 10.3390/e23111404

**Published:** 2021-10-26

**Authors:** Fude Li, Kang Xue, Xuexi Yi

**Affiliations:** Center for Quantum Sciences and School of Physics, Northeast Normal University, Renmin Street 5268, Changchun 130024, China; lifd283@nenu.edu.cn (F.L.); xuekang@nenu.edu.cn (K.X.)

**Keywords:** nonlinear energy band, nonlinear Berry phase, topological phase transition

## Abstract

Topological physics in optical lattices have attracted much attention in recent years. The nonlinear effects on such optical systems remain well-explored and a large amount of progress has been achieved. In this paper, under the mean-field approximation for a nonlinearly optical coupled boson–hexagonal lattice system, we calculate the nonlinear Dirac cone and discuss its dependence on the parameters of the system. Due to the special structure of the cone, the Berry phase (two-dimensional Zak phase) acquired around these Dirac cones is quantized, and the critical value can be modulated by interactions between different lattices sites. We numerically calculate the overall Aharonov-Bohm (AB) phase and find that it is also quantized, which provides a possible topological number by which we can characterize the quantum phases. Furthermore, we find that topological phase transition occurs when the band gap closes at the nonlinear Dirac points. This is different from linear systems, in which the transition happens when the band gap closes and reopens at the Dirac points.

## 1. Introduction

The topological concepts developed in condensed matter theory have a profound impact on physics [1,2,3,4,5,6,7]. They have been now extended to study a variety of photonic structures and, in turn, invoke a wide range of interest in condensed matter physics [8,9,10,11,12]. For systems with interactions, discussions on topological invariant [13,14,15] have become an emerging topic, since the topological classification for systems of single particle was studied extensively [16,17]. In these studies, topological invariants play an important role; for example, quantized Hall conductances [18] are represented in terms of Chern numbers associated with the Berry phase [19,20,21,22], while its extension to the case of four-dimensional quantum Hall conductances is described by second Chern numbers [23].

Thanks to the development of emerging topological photonics [24,25,26], we can theoretically predict and experimentally observe many curious phenomena in optical systems, many of which are caused by nonlinearity [27,28,29,30,31]. For instance, a nonreciprocal quantum-limited amplifier was designed in arrays of coupled resonators [32]; topological polaritons and excitons in strong coupled microcavities [33,34,35] were proposed to create an optomechanical system with Kerr nonlinearities [36,37]; and nonlinear arrays of waveguides [38,39] were suggested for the observation of topological edge states. In such systems, optical nonlinearities are closely related to the remarkable features of topological photonics, resulting in novel observations being offered by strongly correlated states similar to the fractional quantum Hall effect in fermion systems. Stimulated by the potential applications of photonic materials, topological quantum matter and topological transitions in such systems have been widely explored and attracted increasing attention [40,41,42,43]. The theoretical technique for such systems is often within the mean-field approximation, which reduces any many-body problem into an effective one-body problem and can be applied to a number of physical systems to study phenomena such as energy dispersion and phase transitions. In the mean-field approximation, the discussions on the nonlinear problem and the system solutions are reduced to nonlinear Schrödinger equation(NSE) with state-dependent Hamiltonian, which is widely used to study various topics, such as the Bose–Einstein condensate [44,45].

**Figure 1:**
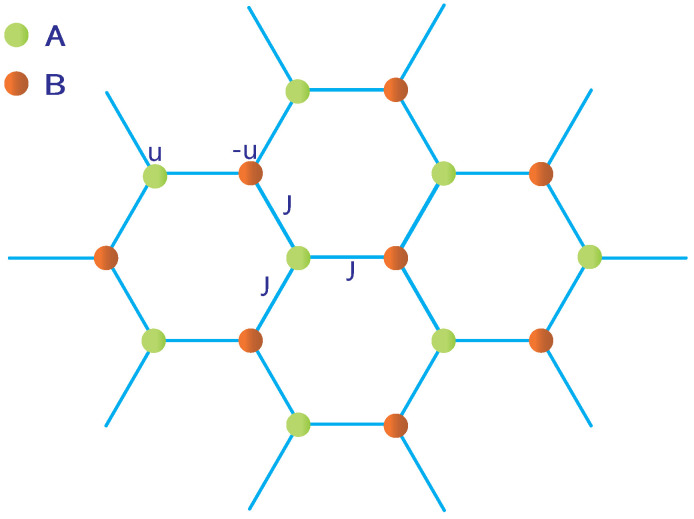
Coupled bosonic hexagonal lattice. Here green (orange) dots denote lattice A(B), on-site potential energy for lattice A(B) is *u*(−u), *J* is the hopping strength between lattice A and lattice B, and the nonlinear on-site interaction constant of the lattice A(B) is κ. $\kappa_{12}$ denotes site–site nonlinear coupling constant between lattice A and lattice B.

In this paper, we aim to study a bosonic version of hexagonal lattice model [46] with nonlinear couplings (see Figure 1) by NSE. This interesting, two-dimensional hexagonal lattice was first purposed in condensed matter physics [47,48] and is attractive for both experimental and theoretical study due to the novel features of the system. The hexagonal lattice can be experimentally realized in graphene, which is a popular system at present and an amazing material in the frontier of physics and technology. Theoretically, the hexagonal lattice is also amazing because it has two sites per unit cell (say, A-site and B-site), so this system can be described by the two-band model. Earlier studies [49,50] have shown that a self-crossing loop would exist in one-dimensional system and a nonlinear two-band model would develop a looped band structure. In the following, we will discuss the dependence of the looped structure [51,52] on the parameters of the system, and shed more light on the nonlinear Dirac cone (NDC) [50] in this two-dimensional system. We find that there are two NDCs in the Brillouin Zone (BZ), and the phase acquired by adiabatically transporting the system on the lowest band around the two NDCs in the BZ is zero, while the phase acquired in the same processing is quantized around only one of the NDCs. Taking the nonlinear effect into account, we analytically calculate the Berry phase and find that the Berry phase is exactly the Aharonov-Bohm phase in the 2D system. The quantized Berry phase is reminiscent of the AB phase [50] and might be taken as a topological invariant for topological materials.

This paper is organized as follows. In Section 2, we introduce a Hamiltonian to describe the hexagonal lattice with effective nonlinear couplings. The NDCs and teh effective Hamiltonian around the NDCs are given and discussed in this section. The realization of this model in an experiment, for example, in a two-dimensional hexagonal array of waveguides with refractive indices, nearest neighbor couplings and on-site Kerr nonlinearity [53], is also suggested. In Section 3, we calculate the Berry phase acquired when the system adiabatically transports around the NDCs of a two-dimensional system. In Section 4, taking the nonlinearity into account, we numerically calculate the Aharonov-Bohm(AB) phase acquired in an adiabatic evolution. We find that the Berry phase is exactly the AB phase in this situation. This suggests that there is no difference between linear and nonlinear systems at this point. In Section 5, we discuss the effect of extra nonlinear interactions on the feature of the system, and examine the robustness of NDCs against small perturbations and study the effect of random noise on the quantized phases. The dependance of the AB phase on the site–site nonlinear coupling strength κ and the on-site potential *u* is also studied in this section. Finally, in Section 6, we summarize our results.

## 2. Nonlinear Dirac Cones and Dynamical Stability

In this section, we first present a derivation for a non-linear two-band model, then we discuss the Dirac cones and dynamical stability of this system. We start with the realization of the Bose–Hubbard model in an optical lattice [54], which can be described by the following Hamiltonian,

(1) $$\begin{array}{ccc} \hat{H} & = & \int dx\int dy{\hat{\Psi }}^{†}\left(x,y\right)\left[-\frac{\hbar^{2}}{2m}\frac{\partial^{2}}{\partial x^{2}}-\frac{\hbar^{2}}{2m}\frac{\partial^{2}}{\partial y^{2}}+V_{external}\left(x,y\right)\right]\hat{\Psi }\left(x,y\right)\\ & + & \frac{1}{2}\int dx\int dy\int dx^{\prime }\int dy^{\prime }{\hat{\Psi }}^{†}\left(x,y\right){\hat{\Psi }}^{†}\left(x^{\prime },y^{\prime }\right)V_{internal}\left(x-x^{\prime },y-y^{\prime }\right)\hat{\Psi }\left(x^{\prime },y^{\prime }\right)\hat{\Psi }\left(x,y\right), \end{array}$$

where $\hat{\Psi }\left(x,y\right)$ is the bosonic field operator, which can annihilate a boson at position (x,y). If we expand this with the Wannier basis $\hat{\Psi }\left(x,y\right)=\sum_{l,m}{\hat{c}}_{l,m}w\left(x-x_{l},y-y_{m}\right)$, where operator ${\hat{c}}_{l,m}$ is defined to annihilate a particle in the Wannier state $w(x-x_{l},y-y_{m})$. To shed light on the effect of nonlinearity, we add on-site nonlinear interactions into the system and assume two-body interaction potential $V_{internal}\left(x-x^{\prime },y-y^{\prime }\right)=\kappa \delta \left(x-x^{\prime },y-y^{\prime }\right)$ with κ denoting the interaction strength [55,56]. We further assume that only nearest-neighbor hopping can occur and consider terms with on-site interactions in terms of their Wannier states. In experiments, this optical external tilted potential in a 2D hexagonal lattice including two sublattices, A and B, can be formed by three retro-reflected laser beams, and potential in sublattices A and B is adjustable, with a phase between different beams [57,58]. Loading bosons (BECs [55,59] is better) into this optical hexagonal lattice, we obtain a bosonic hexagonal lattice model with two different lattices, denoted by A and B, respectively (see Figure 1). Here, we need to replace ${\hat{c}}_{l,m}$ with ${\hat{a}}_{l,m},{\hat{b}}_{l,m}$. The site–site nonlinear couplings between lattices A and B will be discussed later. With this consideration, the system Hamiltonian reads,

(2) $$\begin{array}{ccc} \hat{H} & = & \sum_{l,m}u_{a}{\hat{a}}_{l,m}^{†}{\hat{a}}_{l,m}+u_{b}{\hat{b}}_{l,m}^{†}{\hat{b}}_{l,m}\\ & + & \sum_{l,m}J\left({\hat{a}}_{l,m}^{†}{\hat{b}}_{l,m}+{\hat{a}}_{l,m}^{†}{\hat{b}}_{l-1,m}+{\hat{a}}_{l,m}^{†}{\hat{b}}_{l,m-1}\right)+h.c.\\ & + & \sum_{l,m}\kappa /2\left({\hat{a}}_{l,m}^{†}{\hat{a}}_{l,m}^{†}{\hat{a}}_{l,m}{\hat{a}}_{l,m}+{\hat{b}}_{l,m}^{†}{\hat{b}}_{l,m}^{†}{\hat{b}}_{l,m}{\hat{b}}_{l,m}\right), \end{array}$$

where (l,m) labels the lattice site(l,m), which is an abbreviated representation of coordinates, $r_{A(B)}\left(l,m\right)=lc_{1}+mc_{2}$, $r_{A}\left(l,m\right)=r_{B}\left(l,m\right)+c_{0}$, with $c_{1}=\left(1/2\right)\left[3,\sqrt{3}\right],c_{2}=\left(1/2\right)\left[3,-\sqrt{3}\right]$ and $c_{0}=\left[-1,0\right]$. $a{\left(b\right)}_{l,m}^{†}$($a{\left(b\right)}_{l,m}$) is the creation (annihilation) operators of bosons on the lattice site (l,m) for A(B), $u_{a}$ and $u_{b}$ denotes the tilted on-site potential, *J* is the hopping strength between lattice A and lattice B, and κ stands for the nonlinear interaction strength, which is limited to positive throughout this paper.

Using the Heisenberg equation $i\partial_{t}{\hat{a}}_{l,m}\left({\hat{b}}_{l,m}\right)=\left[{\hat{a}}_{l,m}\left({\hat{b}}_{l,m}\right),\hat{H}\right]$, we can obtain a time-dependent NSE in the real space regarding ${\hat{a}}_{l,m}\left({\hat{b}}_{l,m}\right)$,

(3) $$\begin{array}{ccc} i\partial_{t}{\hat{a}}_{\left(l,m\right)} & = & u_{a}{\hat{a}}_{\left(l,m\right)}+J{\hat{b}}_{\left(l,m\right)}+J{\hat{b}}_{\left(l-1,m\right)}\\ & + & J{\hat{b}}_{\left(l,m-1\right)}+\kappa {\hat{a}}_{\left(l,m\right)}^{†}{\hat{a}}_{\left(l,m\right)}{\hat{a}}_{\left(l,m\right)},\\ i\partial_{t}{\hat{b}}_{\left(l,m\right)} & = & u_{b}{\hat{b}}_{\left(l,m\right)}+J{\hat{a}}_{\left(l,m\right)}+J{\hat{a}}_{\left(l+1,m\right)}\\ & + & J{\hat{a}}_{\left(l,m+1\right)}+\kappa {\hat{b}}_{\left(l,m\right)}^{†}{\hat{b}}_{\left(l,m\right)}{\hat{b}}_{\left(l,m\right)}. \end{array}$$

And then we take the expectation value with Glauber coherent states $\langle \Phi |{\hat{a}}_{l,m}\left({\hat{b}}_{l,m}\right)|\Phi \rangle$ where this coherent state has the form, $|\Phi \rangle ={⨂}_{\left(l,m\right)}|\alpha {\left(\beta \right)}_{l,m}\rangle$, where ${|\alpha \left(\beta \right)}_{l,m}\rangle =e^{{-|\alpha \left(\beta \right)}_{l,m}{|}^{2}/2}$$\sum_{n=0}^{\infty }\frac{{\left(\alpha {\left(\beta \right)}_{l,m}\right)}^{n}}{\sqrt{n!}}|n\rangle$. We get the coherent state amplitude $\alpha {\left(\beta \right)}_{l,m}=\langle \Phi |{\hat{a}}_{l,m}\left({\hat{b}}_{l,m}\right)|\Phi \rangle$ in the time-dependent form of NSE,

(4) $$\begin{array}{ccc} i\partial_{t}\alpha_{\left(l,m\right)} & = & u_{a}\alpha_{\left(l,m\right)}+J\beta_{\left(l,m\right)}+J\beta_{\left(l-1,m\right)}\\ & + & J\beta_{\left(l,m-1\right)}+\kappa {\left|\alpha_{\left(l,m\right)}\right|}^{2}\alpha_{\left(l,m\right)},\\ i\partial_{t}\beta_{\left(l,m\right)} & = & u_{b}\beta_{\left(l,m\right)}+J\alpha_{\left(l,m\right)}+J\alpha_{\left(l+1,m\right)}\\ & + & J\alpha_{\left(l,m+1\right)}+\kappa {\left|\beta_{\left(l,m\right)}\right|}^{2}\beta_{\left(l,m\right)}, \end{array}$$

where we consider equation of motion in the mean-field approximation [60]. By the virtue of periodical boundary condition (PBC) $\alpha {\left(\beta \right)}_{l,m}=\psi_{1(2)}\left(k_{x},k_{y}\right)e^{i(lk_{x}+mk_{y})}$ and within the mean-field approximation [61,62], the time-independent NSE in the momentum space takes the following form,

(5) $$\begin{array}{c} H\left(k_{x},k_{y}\right)\psi \left(k_{x},k_{y}\right)=E\left(k_{x},k_{y}\right)\psi \left(k_{x},k_{y}\right), \end{array}$$

with

(6) $$\begin{array}{c} H\left(k_{x},k_{y}\right)=\left(\begin{array}{cc} u_{a}+\kappa {\left|\psi_{1}\right|}^{2} & d_{x}-id_{y}\\ d_{x}+id_{y} & u_{b}+\kappa {\left|\psi_{2}\right|}^{2} \end{array}\right), \end{array}$$

where we obtain the mean-field Hamiltonian. To make this mathematical form more intuitive, we can transform different on-site potentials $u_{a}$, $u_{b}$ in A and B into potential values with the opposite sign, where $u_{a}\sigma_{+}+u_{b}\sigma_{-}=\left[\left(u_{a}-u_{b}\right)/2\right]\sigma_{z}+\left[\left(u_{a}+u_{b}\right)/2\right]\sigma_{0}$ with $\sigma_{+}=\left[1,0;0,0\right]$, $\sigma_{-}=\left[0,0;0,1\right]$, and $\sigma_{x,y,z}$, $\sigma_{0}$ are Pauli matrices and identity matrix, respectively. For convenience, we set $d_{z}=u=\left(u_{a}-u_{b}\right)/2$ and $u_{a}=-u_{b}$. We obtain,

(7) $$\begin{array}{ccc} & & d_{x}=J\cos k\cdot c_{0}+J\cos k\cdot {ȷ}_{1}+J\cos k\cdot {ȷ}_{2},\\ & & d_{y}=J\sin k\cdot c_{0}+J\sin k\cdot {ȷ}_{1}+J\sin k\cdot {ȷ}_{2},\\ & & d_{z}=u, \end{array}$$

with ${ȷ}_{1}=c_{0}+c_{1}$, ${ȷ}_{2}=c_{0}+c_{2}$, $k=[k_{x},k_{y}]$, and $k_{x}\left(k_{y}\right)$ is the quasimomentum. $\psi \left(k_{x},k_{y}\right)={\left[\psi_{1}\left(k_{x},k_{y}\right),\psi_{2}\left(k_{x},k_{y}\right)\right]}^{T}$ denotes complex amplitudes in the momentum space, and we should address whether these complex amplitudes represent the eigenstate of the mean-field Hamiltonian, so the system is nonlinear since the Hamiltonian is state dependent.

In the following, we will first numerically calculate and plot the energy band of this nonlinear Hamiltonian, and show that around points $k_{1}=\frac{2\pi }{3}\left(1,\frac{1}{\sqrt{3}}\right)$ and $k_{2}=\frac{2\pi }{3}\left(1,-\frac{1}{\sqrt{3}}\right)$, more than two eigenvalues will appear; these two special points are called nonlinear Dirac points [50], and nonlinear Dirac cones (NDCs) can arise around them. Finally, in this section, we analyze the dynamical stability of the system and derive a effective Hamiltonian around the points. Throughout this paper, we assume that all physical variables, including *J*, κ, *u*, time *t*, are of dimensionless, and ℏ=1.

**Figure 2:**
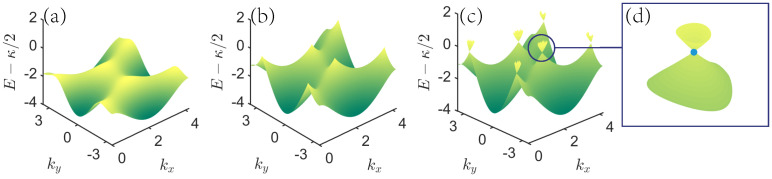
The cone structure in the nonlinear bosonic hexagonal lattice model. Here, we only plot the first two energy bands. The chosen parameters are J=1,u=1.5, (**a**) κ=0, (**b**) κ=3, (**c**) κ=5. The blue circle in (**b**) indicates that a cone structure will be produced. (**d**) Enlarged structure focusing on the cone, the blue dot in this structure indicates nonlinear Dirac points.

Figure 2 shows the first two energy band structures. In the previous studies of a nonlinear system, some loop structures emerge in the energy spectrum [52]. In our case, when the κ=0, the system gets back to the linear one, which is an ordinary hexagonal lattice with mass term *u* [46]. The ground energy band of this linear system is depicted in Figure 2a. As κ gradually increases to a critical value $\kappa_{c}$, specifically, for $\kappa =\kappa_{c}$, the ground energy band starts to produce a cone; see Figure 2b. This critical value $\kappa_{c}$ is related to on-site potential *u*, and we can obtain this relation from the approximate Hamiltonian in the following. For $\kappa >\kappa_{c}$, the cone structure appears [50], and we find the following difference between linear and nonlinear lattice models. Dirac points in the linear lattice models can close and reopen, which corresponds to gapless points and gapped points as parameters change. However, this cannot happen in our nonlinear lattice models. Namely, the nonlinear Dirac points fundamentally change their features, which are gapless points for as long as the parameters reach, and reach beyond, the critical value, at which point no reopening happens. Figure 2c shows the second energy band produced from the ground energy band. This observation is useful for the calculations in Section 3 for the Berry phase in nonlinear systems, and suggests that we need a new topological invariant to witness the difference based on κ in the ground energy band. In other words, in our lattice model with κ=0, Chern number of the lowest band can distinguish different topological phases, but it does not work for $\kappa \geq \kappa_{c}$. This means we need a new topological invariant to characterize the topological phase in this case. In the following, we will propose to use the Berry phase around nonlinear Dirac point as the topological invariant and focus on the first two energy bands and calculate the topological invariant for the ground energy band.

We have already shown the energy bands; however, we do not know whether these energy bands (eigenstates of the Hamiltonian) are stable in the presence of nonlinear interactions. For nonlinear systems, solution stability is very important and if these solutions are not stable, the Berry phase calculated in the next section is meaningless. For the hexagonal lattice model with nonlinear interactions, the stability of eigenstates can be analyzed by the method below, in the same manner as in [63] to analyze a 1D nonlinear system. As we focus on the first two bands, which possess NDCs, we only provide a stability analysis of these two bands.

To be specific, we add a small variation, described by

(8) $$\begin{array}{c} \delta \psi \left(k_{x},k_{y},t\right)={\left[\delta \psi_{1}\left(k_{x},k_{y},t\right),\delta \psi_{2}\left(k_{x},k_{y},t\right)\right]}^{T}, \end{array}$$

which we can add into the states $\psi \left(k_{x},k_{y},t\right)=\psi \left(k_{x},k_{y}\right)e^{-iE(k_{x},k_{y})t},\;\left(\hbar =1\right)$ to solve the time-dependent NSE with Hamiltonian Equation (6). Writing

(9) $$\begin{array}{c} \delta \psi_{j}\left(k_{x},k_{y},t\right)=e^{-iE_{j}\left(k_{x},k_{y}\right)t}\delta \phi_{j}\left(k_{x},k_{y},t\right), \end{array}$$

with $E_{j}\left(k_{x},k_{y}\right)$ denoting the j−th eigenvalue(the *j*-th energy band) of the system, we have

(10) $$\begin{array}{c} i\frac{\partial }{\partial t}\left(\begin{array}{c} \delta \phi (k_{x},k_{y},t)\\ \delta \phi^{*}\left(k_{x},k_{y},t\right) \end{array}\right)=L\left(\begin{array}{c} \delta \phi (k_{x},k_{y},t)\\ \delta \phi^{*}\left(k_{x},k_{y},t\right) \end{array}\right), \end{array}$$

where,

(11) $$\begin{array}{ccc} \delta \phi (k_{x},k_{y},t) & = & {\left[\delta \phi_{1}\left(k_{x},k_{y},t\right),\delta \phi_{2}\left(k_{x},k_{y},t\right)\right]}^{T},\\ \delta \phi^{*}\left(k_{x},k_{y},t\right) & = & {\left[\delta \phi_{1}^{*}\left(k_{x},k_{y},t\right),\delta \phi_{2}^{*}\left(k_{x},k_{y},t\right)\right]}^{T}, \end{array}$$

as well as,

(12) $$\begin{array}{c} L=\left(\begin{array}{cc} H_{s}+H_{1} & H_{2}\\ -H_{2}^{*} & -H_{s}^{*}-H_{1} \end{array}\right), \end{array}$$

with

(13) $$\begin{array}{c} H_{s}=H\left(k_{x},k_{y},\psi \left(k_{x},k_{y}\right)\right)-E\left(k_{x},k_{y}\right)I, \end{array}$$

and

(14) $$\begin{array}{ccc} H_{1} & = & \kappa \left(\begin{array}{cc} |\psi_{1}\left(k_{x},k_{y}\right){|}^{2} & 0\\ 0 & |\psi_{2}\left(k_{x},k_{y}\right){|}^{2} \end{array}\right),\\ H_{2} & = & \kappa \left(\begin{array}{cc} \psi_{1}^{2}\left(k_{x},k_{y}\right) & 0\\ 0 & \psi_{2}^{2}\left(k_{x},k_{y}\right) \end{array}\right). \end{array}$$

**Figure 3:**
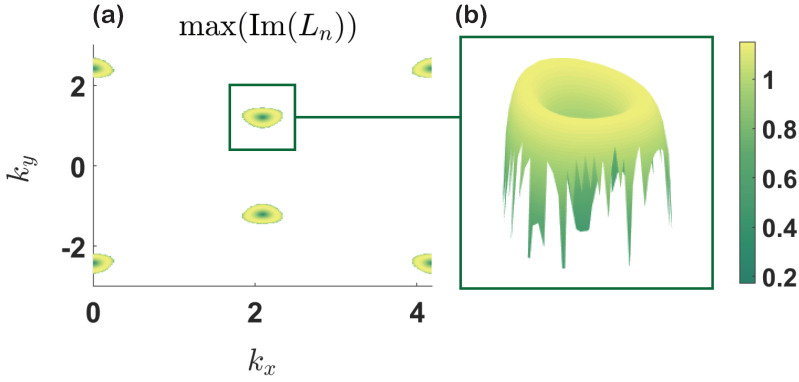
max(Im($L_{n}$)) for the second energy band as a function of $k_{x}$ and $k_{y}$. (**a**) shows that the second energy band is unstable, while the ground energy band is stable (not shown here). (**b**) is an enlarged part of the area where the eigenvalues $L_{n}$ are complex (not real). The chosen system parameters are J=1, u=1.5 and κ=5.

Here, *I* represents identity matrix. Since the variation and its conjugate partner are coupled, we need to add the conjugate variation to the equation. The time evolution of the variation is governed by the operator $e^{-iLt}$, which is not unitary; hence, the eigenvalues $L_{n}$ of *L* can be complex. By examining the eigenvalues of *L*, we can obtain information about the stability of the eigenstates $\psi_{j}\left(k_{x},k_{y}\right)$, j=1,2. This means the system is stable against small variations if the imaginary part of $L_{n}$ ( for ∀n) is zero [64]. We have performed extensive numerical simulations, and the numerical calculation shows that the ground energy band is dynamically stabile throughout the BZ, while the second energy band is unstable, see Figure 3, where we demonstrate the maximal imaginary value of $L_{n}$ for the second band.

Before closing this section, we present an approximate Hamiltonian to describe the system close to the nonlinear Dirac points. The Hamiltonian is valid up to the first order in nonlinear Dirac points. To this end, we write $E=E^{\left(0\right)}+E^{\left(1\right)}$, $d_{i}=d_{i}^{\left(0\right)}+d_{i}^{\left(1\right)},i=x,y,z$, where superscripts (0) and (1) represents zero-order and first-order in nonlinear Dirac points, respectively. Around the nonlinear Dirac points $k_{1}=\frac{2\pi }{3}\left(1,\frac{1}{\sqrt{3}}\right),k_{2}=\frac{2\pi }{3}\left(1,-\frac{1}{\sqrt{3}}\right)$, we obtain an approximate Hamiltonian as

(15) $$\begin{array}{c} H\left(k_{x},k_{y}\right)=d_{x}^{\left(1\right)}\sigma_{x}+d_{y}^{\left(1\right)}\sigma_{y}-\frac{2uE^{\left(1\right)}}{\kappa }\sigma_{z}+\frac{\kappa }{2}\sigma_{0}, \end{array}$$

where

(16) $$\begin{array}{ccc} d_{x}^{\left(1\right)} & = & -\frac{3\sqrt{3}}{4}J\left(k_{x}-\frac{2\pi }{3}\right)\mp \frac{3}{4}J\left(k_{y}\mp \frac{2\sqrt{3}\pi }{9}\right),\\ d_{y}^{\left(1\right)} & = & \frac{3}{4}J\left(k_{x}-\frac{2\pi }{3}\right)\mp \frac{3\sqrt{3}}{4}J\left(k_{y}\mp \frac{2\sqrt{3}\pi }{9}\right),\\ E^{\left(1\right)} & = & \pm \kappa \sqrt{\frac{{\left(d_{x}^{\left(1\right)}\right)}^{2}+{\left(d_{y}^{\left(1\right)}\right)}^{2}}{\kappa^{2}-4u^{2}}}. \end{array}$$

The first two energy bands are described by different Hamiltonian because the hexagonal lattice Hamiltonian is eigenstate-dependent; hence, it is nonlinear. It should be worth noting that this approximate Hamiltonian would lead to the appearance of nonlinear Dirac points($\kappa >\kappa_{c}$); meanwhile, we find that $E^{\left(0\right)}$ is real when κ>2u so that we obtain more than two eigenvalues; thus, $\kappa_{c}=2u$ is the critical point for appearance of NDCs.

## 3. Nonlinear Berry Phase

In this section, we will follow the idea in Ref. [65] to calculate the Berry phase [66,67] acquired when the system adiabatically transports around the nonlinear Dirac points. The idea is as follows. We assume that k is the parameter vector that changes slowly with time. Introducing an adiabatic parameter $\epsilon ∼\frac{dk}{dt}$ that can be viewed as the measure of how slowly the parameters change and writing $\psi \left(k\left(t\right)\right)=e^{-i\Lambda }\phi \left(k\left(t\right)\right)$, with ϕ(k(t)) belonging to the projective Hilbert space, we can expand ϕ(k(t)) and Λ in power of ϵ [63,65],

(17) $$\begin{array}{ccc} \phi (k(t)) & = & \epsilon^{0}\phi^{\left(0\right)}\left(k\left(t\right)\right)+\epsilon^{1}\phi^{\left(1\right)}\left(k\left(t\right)\right)+\cdots ,\\ \Lambda & = & \Lambda^{\left(0\right)}\left(\epsilon^{0}\right)+\Lambda^{\left(1\right)}\left(\epsilon^{1}\right)+\cdots \;. \end{array}$$

When the adiabatic parameter ϵ tends towards zero, the adiabatic limit can be achieved. Substituting this expansion into the time-dependent NSE, we obtain,

(18) $$\begin{array}{c} \frac{d\Lambda }{dt}=\gamma^{\left(0\right)}\left(\epsilon^{0}\right)+\gamma^{\left(1\right)}\left(\epsilon^{1}\right)+\cdots \;. \end{array}$$

In the adiabatic limit, $\phi^{\left(0\right)}\left(k\left(t\right)\right)$ is the instantaneous eigenstate and $\phi^{\left(1\right)}\left(k\left(t\right)\right)$ is induced by the slow change, which depends on the adiabatic parameter ϵ and is proportional to ϵ. After simple algebras, we have,

(19) $$\begin{array}{ccc} \gamma^{\left(0\right)}\left(\epsilon^{0}\right) & = & E^{\left(0\right)}\left(k\left(t\right)\right),\\ \gamma^{\left(1\right)}\left(\epsilon^{1}\right) & = & \sum_{\alpha =1,2}-i{\left(\phi_{\alpha }^{\left(0\right)}\right)}^{*}\frac{\partial }{\partial t}\phi_{\alpha }^{\left(0\right)}+\kappa {\left|\phi_{\alpha }^{\left(0\right)}\right|}^{2}C_{\alpha }. \end{array}$$

Here, $\phi_{\alpha }^{\left(0\right)}$ and $\phi_{\alpha }^{\left(1\right)}$ represent the element of vector $\phi^{\left(0\right)}\left(k\left(t\right)\right)$ and $\phi^{\left(1\right)}\left(k\left(t\right)\right)$, respectively. $C_{\alpha }={\left(\phi_{\alpha }^{\left(0\right)}\right)}^{*}\phi_{\alpha }^{\left(1\right)}+\phi_{\alpha }^{\left(0\right)}{\left(\phi_{\alpha }^{\left(1\right)}\right)}^{*}$ was defined, which can be obtained by solving the following equations,

(20) $$\begin{array}{ccc} \sum_{\alpha =1,2}C_{\alpha } & = & 0,\\ \sum_{\beta =1,2}g\phi_{\alpha }^{\left(0\right)}{\left|\phi_{\beta }^{\left(0\right)}\right|}^{2}C_{\beta }-g\phi_{\alpha }^{\left(0\right)}C_{\alpha } & = & \sum_{\beta =1,2}i\left\{\phi_{\alpha }^{\left(0\right)}\left[{\left(\phi_{\beta }^{\left(0\right)}\right)}^{*}\frac{\partial }{\partial t}\phi_{\beta }^{\left(0\right)}\right]-\frac{\partial }{\partial t}\phi_{\alpha }^{\left(0\right)}\right\},\;\alpha =1,2. \end{array}$$

The equation on the first line of Equation (20) was derived from the normalization condition, $\phi^{†}\phi =1$, with $\phi =\phi^{\left(0\right)}+\phi^{\left(1\right)}$ and setting ${\left(\phi^{\left(0\right)}\right)}^{†}\phi^{\left(0\right)}=1$. The second line was obtained by substituting $\phi =\phi^{\left(0\right)}+\phi^{\left(1\right)}$ into the time-dependent NSE with $\phi^{\left(0\right)}$ being the solution of the time-independent NSE.

Now, we apply this theory to our system. First, we calculate the Berry phase by using the eigenfunctions solved by the approximate Hamiltonian in Equation (15), acquired around nonlinear Dirac points in the lower branch for κ>2u. For κ<2u, the approximate eigenfunctions can be obtained in the same manner. These eigenfunctions read, for κ<2u,

(21) $$\begin{array}{c} \Phi =\left(\begin{array}{c} \phi_{1}^{\left(0\right)}\\ \phi_{2}^{\left(0\right)} \end{array}\right)=\left(\begin{array}{c} \cos\frac{\theta }{2}\\ \sin\frac{\theta }{2}e^{i\varphi } \end{array}\right), \end{array}$$

with

(22) $$\begin{array}{ccc} \tan\varphi (k_{x},k_{y}) & = & \frac{d_{y}^{\left(1\right)}}{d_{x}^{\left(1\right)}},\\ \tan\theta (k_{x},k_{y}) & = & \sqrt{\frac{{\left(d_{x}^{\left(1\right)}\right)}^{2}+{\left(d_{y}^{\left(1\right)}\right)}^{2}}{u}}, \end{array}$$

where $d_{x}^{\left(1\right)}$ and $d_{y}^{\left(1\right)}$ are defined in Equation (16), and ground energy $E=E^{\left(0\right)}+E^{\left(1\right)}$, with

(23) $$\begin{array}{c} E^{\left(1\right)}=\frac{D_{0}-\sqrt{D_{1}\left({\left(d_{x}^{\left(1\right)}\right)}^{2}+{\left(d_{y}^{\left(1\right)}\right)}^{2}\right)+D_{2}}}{F}, \end{array}$$

with,

(24) $$\begin{array}{ccc} D_{0} & = & 4u^{3}-4\kappa u^{2}+4\kappa^{2}u,\\ D_{1} & = & 4\left(2u-\kappa \right)\left(10u-\kappa \right)u^{2},\\ D_{2} & = & {\left(2u-\kappa \right)}^{4}u^{2},\\ F & = & 20u^{2}-12\kappa u+\kappa^{2}. \end{array}$$

Using the expression derived for Λ as well as the equation of $C_{\alpha }$ in the last section, we have,

(25) $$\begin{array}{ccc} \Lambda^{\left(1\right)} & = & \oint_{\Gamma }dk\sum_{\alpha =1,2}-i{\left(\phi_{\alpha }^{\left(0\right)}\right)}^{*}\partial_{k}\phi_{\alpha }^{\left(0\right)}+\kappa {\left|\phi_{\alpha }^{\left(0\right)}\right|}^{2}C_{\alpha }\\ & = & \pi \left(1-\cos\theta \right)+\oint_{\Gamma }dk\frac{1}{2}\cos^{2}\theta C_{1}+\frac{1}{2}\sin^{2}\theta C_{2}\\ & = & 0, \end{array}$$

where $C_{1}=C_{2}=0$, θ→0 when κ<2u, and we set a closed small loop, denoted by Γ in the BZ, enclosing the suspicious nonlinear Dirac points. We find that tanθ∼Γ→0 when this loop is small. As a result, in the Bloch sphere, this small loop reduces to a point located at the north pole in Bloch sphere so that $C_{1}=C_{2}=0$. The physics behind this results show that when κ<2u, the energy band is flat. In other words, there is no singular point on the band, and hence the Berry phase is zero.

The situation changes when κ>2u. The eigenfunction in this case takes the same form as in Equation (21), but with different θ and φ,

(26) $$\begin{array}{c} \tan\varphi (k_{x},k_{y})=\frac{d_{y}^{\left(1\right)}}{d_{x}^{\left(1\right)}},\;\tan\theta =\frac{\sqrt{\kappa^{2}-4u^{2}}}{2u}. \end{array}$$

By the same equation, with $C_{1}=-C_{2}=\frac{1}{2\kappa }\frac{\partial \varphi }{\partial k}$ and θ=const when κ>2u, the Berry phase follows,

(27) $$\begin{array}{ccc} \Lambda^{\left(1\right)} & = & \oint_{\Gamma }dk\sum_{\alpha =1,2}-i{\left(\phi_{\alpha }^{\left(0\right)}\right)}^{*}\partial_{k}\phi_{\alpha }^{\left(0\right)}+\kappa {\left|\phi_{\alpha }^{\left(0\right)}\right|}^{2}C_{\alpha }\\ & = & \pi \left(1-\cos\theta \right)+\oint_{\Gamma }dk\frac{1}{2}\cos^{2}\theta C_{1}+\frac{1}{2}\sin^{2}\theta C_{2}\\ & = & \pi . \end{array}$$

Collecting these results, we obtain a relation between the Berry phase and the nonlinear strength κ,

(28) $$\begin{array}{c} \Lambda^{\left(1\right)}=\left\{\begin{array}{c} \pi ,\kappa >2u\\ 0,\kappa <2u \end{array}\right., \end{array}$$

where the critical value of $\kappa_{c}$ is 2u. Notice that, the phase change at κ=2u is very sharp, ranging from 0 to π.

One may be curious about the Berry phase beyond the approximate Hamiltonian approach. At the end of this section, we will calculate the Berry phase based on the exact eigenstate of the nonlinear Hamiltonian Equation (6), which can be written in the following form,

(29) $$\begin{array}{c} H\left(k\right)=d_{x}\sigma_{x}+d_{y}\sigma_{y}+d_{z}\left(E\right)\sigma_{z}, \end{array}$$

with

(30) $$\begin{array}{c} d_{z}\left(E\right)=u\Delta \left(E\right), \end{array}$$

where we have rewritten the state-dependent Hamiltonian as an energy-dependent Hamiltonian and define

(31) $$\begin{array}{c} \Delta \left(E\right)=\frac{2E(k)-\kappa }{2(E(k)-\kappa )}, \end{array}$$

$d_{x}$, $d_{y}$ are given in Equation (7).

Similar to the last section, we can provide the eigenfunction as before, but with different θ and φ,

(32) $$\begin{array}{c} \tan\varphi (k_{x},k_{y})=\frac{d_{y}}{d_{x}},\;\tan\theta =\frac{\sqrt{d_{x}^{2}+d_{y}^{2}}}{d_{z}\left(E\right)}. \end{array}$$

To make a comparison with the results in the last section, we separately discuss the case of κ<2u and κ>2u. We establish a closed loop enclosing the nonlinear Dirac point $k_{1}\left(k_{2}\right)$ in the BZ, which is a gapless point when κ>2u. Meanwhile, we obtain $E(k_{1}\left(k_{2}\right))$ whose value is κ−u when κ<2u and κ/2 when κ>2u. This implies that if κ>2u, the closed loop would enclose a gapless point localized at $k_{1}\left(k_{2}\right)$. As a consequence, we need to obtain a complex connected closed curve to dig up a small circle including this gapless point. Different from κ<2u regime, namely, the case without gapless points, an extra integral of the small circle including gapless point makes a contribution to the Berry phase, so that we obtain π in the Berry phase. As a comparison to κ>2u, the gapless point will disappear in this area leading to a zero Berry phase for κ<2u. We get the same result as in the last section, where we use approximate analytical methods.

## 4. Aharonov-Bohm (AB) Phase Accumulated in Adiabatic Transport

**Figure 4:**
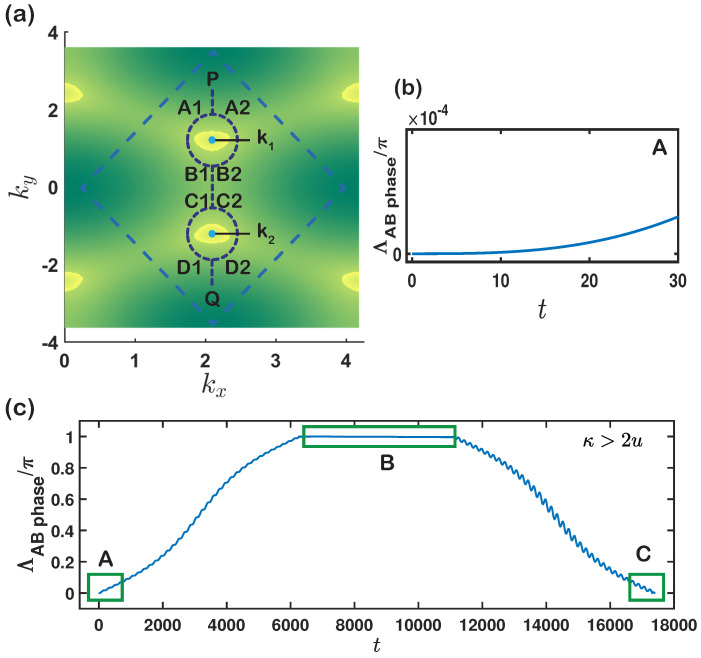
(**a**) Two paths PA1B1C1D1Q and PA2B2C2D2Q that enclose the nonlinear Dirac points. The start and end points are P($k_{1x}$,$k_{1y}$+R+r) and Q($k_{2x}$,$k_{2y}$-R-r), respectively, where $r=10^{-4}$. Dashed blue circles indicate the position of NDCs, and blue dotes label the nonlinear Dirac points. Dashed blue lines are the common paths and the dashed square denotes the Brillouin Zone(BZ). (**b**) is enlarged area of A in (**c**), which show that the phase difference in the initial stage is zero and it finally comes back to zero. (**c**) Phase difference between two adiabatic paths versus time *t*. All parameters are chosen to be the same as before, J=1, u=1.5, κ=5 and $\omega_{l}=-0.5\times 10^{-3}$.

In this section, we will examine how phase difference accumulates when the system transports adiabatically along two paths. The two paths are depicted in Figure 4a and the phase difference can be viewed as the Aharonov-Bohm (AB) phase [68] in momentum space [63]. This consideration is reminiscent of interference setup [69] in momentum space, which might pave the way for the completion of experimentally topological optical lattice systems. By virtue of this kind of setup, we can obtain the nonlinear Berry phase from numerical results based on time-dependent NSE.

We restrict the study in the adiabatic limit. For this purpose, we use a slow variation in angle dφ/dt, which will be defined below to quantify the adiabatic evolution. Numerically, we can check whether the system state is very close to the instantaneous eigenstate |E(t)〉 of H(t).

The curves used to observe the phase difference in a complete adiabatic evolution process are shown in Figure 4a. At the beginning of this process, these two time-dependent states will start to separate from each other (point P in Figure 4a), and then they will simultaneously experience two semicircles, including a nonlinear Dirac point and a straight line (B1C1 or B2C2), which connects two semicircles. Next, they will reunite so that the AB phase will be generated by these two paths in the momentum space (point Q in Figure 4a). This circumstance is reminiscent of the Aharonov-Bohm effect.

To be specific, we choose the two paths of $k_{x}\left(t\right)=k_{1(2)x}+R\cos\varphi (t)$ and $k_{y}\left(t\right)=k_{1(2)y}+R\sin\varphi (t)$, where $k_{1(2)x},k_{1(2)y}$ represents the positions of nonlinear Dirac points. $R=10^{-4}$ and ω=dφ/dt is a small parameter to enable the adiabatic evolution. Two cases are considered: one is a full circle enclosing one of the NDCs as an A1B1 path and A2B2 path, depicted in Figure 4a. The other is two full circles enclosing all NDCs in BZ as A1B1C1D1 path and A2B2C2D2 path in Figure 4a. We chose the total evolution time $T=2\pi \times 10^{3}$ in the first case and $T=4\pi \times 10^{3}+\left(k_{y02}-k_{y01}+2R\right)/\omega_{l}$, with $\omega_{l}$ being the variance rate in B1C1 and B2C2 in the second case. The time step is set as $dt=T\times 10^{-3}$, and the evolution ends at $\left(k_{x},k_{y}\right)=\left(k_{x}\left(T\right),k_{y}\left(T\right)\right)$, where the phase difference of two paths can be directly located. The phase acquired in the first case is π with nonlinear Dirac points, while it is zero in the absence of nonlinear Dirac points, see Figure 4.

For κ>2u, the phase acquired in the second case is given in Figure 4c, where an oscillation can be found in the phase difference. For κ<2u, the phase difference in the whole process is always zero, so we focus on the κ>2u regime. The following observation can be made: (1) The two states are initially set at start point P, and then these two states experience path PA1 and path PA2, respectively, where they do not have a phase difference in the initial paths, as shown in in area A of Figure 4c. (2) These two states travel along path A1B1 and path A2B2, respectively. When they arrive at B1 and B2, the phase difference is π; it remains unchanged in the B1C1 and B2C2 paths, as denoted in area B. (3) These two states reach endpoint point Q and the phase difference returns to zero, as depicted in area C. Finishing the paths in (1) and (2) together can be viewed as the system completing a path that encloses only one NDC, which leads to the quantization of the acquired nonlinear Berry phase on this path. The paths in (2) and (3) comprise a curve enclosing another NDC, resulting in the quantization of the nonlinear Berry phase acquired around another NDC but with teh opposite sign.

## 5. Site-Site Nonlinear Coupling, Perturbation and Noise

In the above analysis, the intersite nonlinear couplings are ignored. In this section, we will analyze the effect of intersite nonlinear couplings and imperfection on the predicted feature. For this goal, we consider how site-site nonlinear coupling influences the quantization of the Berry phase, and examine the robustness of the NDCs against perturbations and noises.

Let us start from the Hamiltonian, describing hexagonal lattices with both intrasite nonlinear couplings κ and intersite nonlinear couplings $\kappa_{12}$ [70,71,72],

(33) $$\begin{array}{ccc} H & = & \sum_{l,m}u_{a}{\hat{a}}_{l,m}^{†}{\hat{a}}_{l,m}+u_{b}{\hat{b}}_{l,m}^{†}{\hat{b}}_{l,m}\\ & + & \sum_{l,m}J\left({\hat{a}}_{l,m}^{†}{\hat{b}}_{l,m}+{\hat{a}}_{l,m}^{†}{\hat{b}}_{l-1,m}+{\hat{a}}_{l,m}^{†}{\hat{b}}_{l,m-1}\right)+h.c.\\ & + & \sum_{l,m}\kappa /2\left({\hat{a}}_{l,m}^{†}{\hat{a}}_{l,m}^{†}{\hat{a}}_{l,m}{\hat{a}}_{l,m}+{\hat{b}}_{l,m}^{†}{\hat{b}}_{l,m}^{†}{\hat{b}}_{l,m}{\hat{b}}_{l,m}\right)\\ & + & \sum_{l,m}\kappa_{12}{\hat{a}}_{l,m}^{†}{\hat{b}}_{l,m}^{†}{\hat{b}}_{l,m}{\hat{a}}_{l,m}. \end{array}$$

Following the same procedure as we did in the first section, we can obtain $E^{\left(1\right)}$ modified by $\kappa_{12}$,

(34) $$\begin{array}{ccc} E^{\left(1\right)} & = & \pm \kappa \left(\kappa -\kappa_{12}\right)\sqrt{\frac{{\left(d_{x}^{\left(1\right)}\right)}^{2}+{\left(d_{y}^{\left(1\right)}\right)}^{2}}{{\left(\kappa -\kappa_{12}\right)}^{2}-4u^{2}}}. \end{array}$$

Similarly, the critical value is given by $\kappa_{c}=\kappa_{12}+2u$, where $\kappa_{12}$ is viewed as an extra constant to change the critical value.

**Figure 5:**
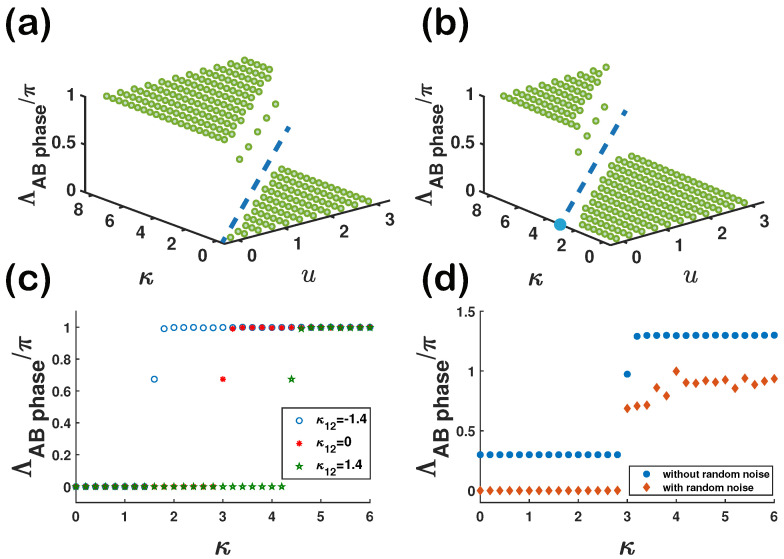
Berry phase versus *u* and κ with (**a**) and without (**b**) the site-site nonlinear couplings. $\kappa_{12}=3$ was chosen for (**b**). The two dashed blue lines denote the boundary (critical lines) for the system to have a 0 or π phase. Clearly, the line is shifted by $\kappa_{12}$ in (**b**). (**c**) When $\kappa_{12}=-1.4,0,1.4$, AB phase versus κ. (**d**) AB phase versus temporal random noise. The strength of the random noise $J_{r}$ is set eas $1.5\times 10^{-4}$. AB phase with temporal random noise is vertically offset for clarity. System parameters are fixed to be the same as before, J=1, u=1.5 in (**c**,**d**).

As shown in Figure 5a, it is necessary to discuss the relationship between the parameters *u* and κ, as well as the phase difference that is obtained. In the noninteracting lattice model, *u* is the mass term that can break down the symmetry in a hexagonal lattice model, and *u* can open and close the band gaps. The nonlinear Dirac points are closed forever if the gap starts to close at $\kappa =\kappa_{c}$. When the NDCs appear $\left(\kappa >\kappa_{c}\right)$, the Berry phase remains quantized, in agreement with the phase difference (also known as the AB phase). It remains quantized in our numerical calculations.

In general, *u* in the linear lattice model can be used to characterize different topological phases, but in the system with nonlinear couplings κ, this does not work. Namely, the system can be in the same topological phase with different values of *u*, while it can be in two distinct phases with the same *u*. Nonlinear Dirac points are especially different from its linear counterparts, in that the energy band gaps close and reopen in the linear case, while the energy gaps close and do not reopen again.

The nonlinear site-site couplings $\kappa_{12}$ can alter the position of topological transition; see Figure 5b. Through the modulation of coupling parameters, such as κ, $\kappa_{12},$ different topological phases can be reached. From Figure 4d, we find that phase difference will come back to zero if the two time-dependent states travel around all the nonlinear Dirac points in the entire BZ, so we anticipate that it is possible for these two time-dependent states to not produce a quantized phase difference in the whole evolution process in this case. In realistic experiment, the two evolution paths may be separately influenced by noise. To examine the effect of noise on the quantization of the phase, we introduce a random Hamiltonian $H_{random}\left(t\right)=J_{r}d_{random}\cdot \sigma$ to the system, where $J_{r}$ denotes the strength of random noise. In this case, the total Hamiltonian is the system Hamiltonian in Equation (6) plus this random Hamiltonian. Here, $d_{random}$ is a randomly created number in the interval, $0<d_{random}<1$. The results can be obtained by averaging over the noises, and are shown in Figure 5d, which suggests that we should keep the whole setup clean and try to avoid any disturbance, because the quantization of the AB phase is sharply changed due to the presence of noise. Thus, we claimed that the quantization of the phase is fragile; this means that the quantization can be easily broken down by small random noises, as in Figure 5d.

From Figure 5c,d (especially (c)) we find that there are values between 0 and 1 around $\kappa_{c}$. When we decrease the step of κ, we find that the phase continuously changes with κ around $\kappa_{c}$.

In topological insulators without nonlinear couplings, DCs are unstable due to their extra mass term. One may wonder, if the stability condition changes when nonlinearity is taken into account. In the following, we will examine that stability by introducing an additional term of perturbation $H_{p}=g_{x}\sigma_{x}+g_{y}\sigma_{y}+g_{z}\sigma_{z}$ into the Hamiltonian Equation (6), we find that these NDCs are robust against perturbations for this 2D system. The same robustness was found in the 1D nonlinear Su–Schrieffer–Heeger(SSH) chain in [63]. The nonlinear Dirac points in our system would be changed by the perturbation. This can be understood by the approximate Hamiltonian with perturbations,

(35) $$\begin{array}{c} H_{a}=\left(d_{x}^{\left(1\right)}+g_{x}\right)\sigma_{x}+\left(d_{y}^{\left(1\right)}+g_{y}\right)\sigma_{y}-\frac{2\left(u+g_{z}\right)E^{\left(1\right)}}{\kappa }\sigma_{z}+\frac{\kappa }{2}\sigma_{0}, \end{array}$$

where

(36) $$\begin{array}{c} E^{\left(1\right)}=\pm \kappa \sqrt{\frac{{\left(d_{x}^{\left(1\right)}+g_{x}\right)}^{2}+{\left(d_{y}^{\left(1\right)}+g_{y}\right)}^{2}}{\kappa^{2}-4{\left(u+g_{z}\right)}^{2}}}. \end{array}$$

**Figure 6:**
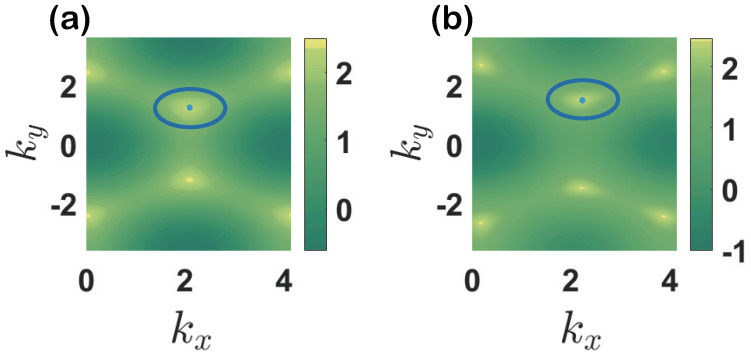
Robustness of NDCs with or without perturbation in (**a**) $g_{x}=0$, $g_{y}=0$, $g_{z}=0$ and (**b**) $g_{x}=0.35$, $g_{y}=0.3$, $g_{z}=0.22$. Blue circles and spots denote position of NDCs and nonlinear Dirac points respectively. Pertubation we choose is arbitrary and system parameters are fixed as before, J=1, u=1.5 and κ=5.

Here, $g_{x}$, $g_{y}$ and $g_{z}$ are perturbation parameters. In the Figure 6, a small shift in the NDCs can be found; they are stable and robust against the perturbations. Note that in the whole analysis, we set the parameters properly to make sure that NDCs exist.

## 6. Discussion and Conclusions

The realization can be briefly summarized as follows. Firstly, our model with on-site nonlinear coupling can be realized in coupled cavity arrays (where atoms and photons are trapped) with on-site interaction [73], where the photons effectively interact with each other inside a cavity via nonlinear medium (for example, Kerr medium). The strength of the nonlinearity can be tuned. Secondly, our model with inter-site nonlinear coupling can be realized in an array of QED cavities coupled with a nonlinear medium, and nonlinear couplings lead to nearest-neighbor Kerr terms [71,72]. In this way, two types of couplings can be characterized by two different parameters.

To summarize, we studied the nonlinear Dirac cone (NDC) in a hexagonal lattice with on-site and site-site nonlinear interactions. Gapped or gapless nonlinear Dirac points were found, with a boundary at the critical point given by $\kappa =\kappa_{c}$. This critical value is changed when the site–site nonlinear coupling is taken into account. The Berry phase acquired for a system transporting adiabatically around the nonlinear Dirac points is calculated, which is π when the system possesses a looped band structure, while it is zero in another case. This phase can be calculated by means reminiscent of the interference in terms of the Aharonov-Bohm phase.

This result suggests that the nonlinear Berry phase in this two-dimensional(2D) system could be a kind of topological number to characterize the topology of 2D systems, as well as it is characterized in 1D systems [63]. This stimulates further studies on the emerging topological photonic structures and topological lasing cavities, such as gyromagnetic photonic crystals, helical waveguides, array of ring waveguides, electric circuits with nonlinear capacitance, etec. We hope that the special structures found in this paper can still exist in the mentioned systems, and maybe we could find some more curious features in these photonic structures. Without a doubt, pursuing these possible research avenues will be the main concern in our future work.

## Abbreviations

The following abbreviations are used in this manuscript:

AB	Aharonov-Bohm
NSE	Nonlinear Schrödinger equation
NDC	Nonlinear Dirac cone
